# Extraction and Application of Laccases from Shimeji Mushrooms (*Pleurotus ostreatus*) Residues in Decolourisation of Reactive Dyes and a Comparative Study Using Commercial Laccase from *Aspergillus oryzae*

**DOI:** 10.4061/2010/905896

**Published:** 2010-11-01

**Authors:** Ricardo Sposina S. Teixeira, Patrícia Maia Pereira, Viridiana S. Ferreira-Leitão

**Affiliations:** ^1^National Institute of Technology, Ministry of Science and Technology, Avenue Venezuela, 82 Centro, 20081-312 Rio de Janeiro, RJ, Brazil; ^2^Laboratory of Enzyme Technology, Chemistry Institute, Federal University of Rio de Janeiro, Avenue Athos da Silveira Ramos 149, Bloco A, Ilha do Fundão, 21949-900 Rio de Janeiro, RJ, Brazil

## Abstract

Oxidases are able to degrade organic pollutants; however, high costs associated with biocatalysts production still hinder their use in environmental biocatalysis. Our study compared the action of a commercial laccase from *Aspergillus oryzae* and a rich extract from *Pleurotus ostreatus* cultivation residues in decolourisation of reactive dyes: Drimaren Blue X-3LR (DMBLR), Drimaren Blue X-BLN (DMBBLN), Drimaren Rubinol X-3LR (DMR), and Drimaren Blue C-R (RBBR). The colour removal was evaluated by considering dye concentration, reaction time, absence or presence of the mediator ABTS (2,2′-azino-bis(3-ethylbenzothiazoline-6-sulfonic acid), and the source of laccase. The presence of ABTS was essential for decolourisation of DMR (80–90%, 1 h) and RBBR (80–90%, 24 h) with both laccases. The use of ABTS was not necessary in reactions containing DMBLR (85–97%, 1 h) and DMBBLN (63–84%, 24 h). The decolourisation of DMBBLN by commercial laccase showed levels near 60% while the crude extract presented 80% in 24 h.

## 1. Introduction

In the textile industry, reactive dyes have been commonly used due to their advantages such as better dyeing processing conditions and bright colours [1]. The reactive dyes also exhibit a wide range of different chemical structures, which are primarily based on substituted aromatic compounds and heterocyclic groups [2]. 

Wastewater from dyeing industries has recalcitrant compounds and presents low biodegradability in conventional biological treatment plants. Biological processes are sensitive to shock loads and require long hydraulic retention times. These processes also form large amounts of solid residues and provide low efficiency in colour removal [3–5]. Earlier studies considered the combination of biological and enzymatic treatment aiming for the reduction of organic matter and decolourisation, respectively [6, 7]. 

White-rot fungi have attracted increasing attention. Many studies showed that their ligninolytic enzymes have the ability to degrade recalcitrant compounds; therefore, they are able to decolourise different classes of industrial dyes [8–15]. *Pleurotus ostreatus* is a white-rot fungus that produces a ligninolytic enzyme complex rich in several laccase isoenzymes [16]. Laccases (EC 1.10.3.2) are phenol oxidases that catalyse one-electron oxidation of many aromatic substrates (polyphenols, methoxy-substituted monophenols, aromatic amines, etc.) with the concomitant reduction of O_2_ to H_2_O [17, 18]. The interest in laccases for biotechnological applications increased with the discovery of their ability to oxidize high-redox-potential substrates in the presence of synthetic mediators [19], which allows an extended substrate range of laccases [20]. The ABTS (2,2′-azino-bis-(3-ethylbenzothiazoline-6-sulfonic acid) is often used as a carrier of electrons in reactions mediated by laccases [14, 16, 18, 21, 22].

Laccases are very promising as environmental biocatalysts, and some studies report dye degradation by these enzymes [16, 23]. Remazol Brilliant Blue R and Drimaren Blue X-3LR are anthracene and diazo dyes, respectively. These classes of reactive dyes are the most used in the textile industry and have been widely employed in many degradation studies [14, 16, 21, 23, 24]. 

The enzymatic treatments are still not commonly used in the textile industries. As mentioned before, the high costs associated with biocatalysts production and application still hinder their use on large scale with environmental purposes. The high cost of importation is the main obstacle to the application of enzymes on large scale. The enzyme extraction from the residues of solid-state fermentation might be one alternative for low-cost enzyme use and consequently environmental biocatalysis consolidation.

Toyobo do Brazil LTDA produces the edible mushroom shimeji (*Pleurotus ostreatus*). This production generates a large volume of colonized residual waste, 300–500 g of residue per 100 g of mushroom produced, which could be used to obtain low-cost ligninolytic enzyme complex. Considering that the consumption of these mushrooms has been increasing annually, due to its nutraceutical properties, it is important to give an appropriate destination to these residues. Additionally, this procedure allows for the utilization of an agroindustrial residue to obtain an added value product and minimize industrial disposal. 

This study aimed at obtaining an alternative and low-cost ligninolytic enzyme pool from an agroresidue from the commercial production of *Pleurotus ostreatus* and compares its utilization with a commercial laccase from *Aspergillus* oryz*ae* in the decolourisation of four textile reactive dyes. Similar study previously reported showed a very promising action of peroxidases from shimeji in decolourisation of RBBR [14]. The value of the present work lies in the identification of significant laccase activity in agrowaste from commercial production of edible mushroom shimeji. Another aspect that also deserves mention is that when comparing the crude extract with the commercial enzyme, the first was more efficient in the degradation of the dyes studied. During the dye degradation study, the following parameters were evaluated: dye concentration, reaction time, absence or presence of ABTS mediator, and the source of laccase (*P. ostreatus *extract or commercial *A. oryzae*). Chemical structures of the dyes studied and their respective wavelength are presented in Table 1.

## 2. Material and Methods

### 2.1. Dyes and Chemicals

Four reactive dyes, Drimaren Blue X-3LR (DMBLR), Drimaren Blue X-BLN (DMBBLN), Drimaren Rubinol X-3LR (DMR), and Drimaren Blue CL-R (RBBR), representing different chemical classes (Table 1) were provided by Maria Candida Textile Industry LTDA, Paracambi, Rio de Janeiro, Brazil. 2,2′-azino-bis(3-ethylbenzothiazoline-6-sulfonic acid) (ABTS—98% purity), 2,4-dichlorophenol, and 4-aminoantipyrine were purchased from Sigma Chemical Co. (St. Louis, MO, USA). Filter paper (FP) (Whatman no. 2) was purchased from GE Healthcare Life Sciences (São Paulo, SP, Brazil). All other chemicals were analytical grade reagents. Commercial laccase produced by a genetically modified *Aspergillus oryzae* was kindly provided by Novozymes (Novozym 51003). This enzyme is a robust stable laccase used for lignin modification in pulp and paper industry.

### 2.2. Enzyme Extraction and Storage Stability

The laccase rich extract was obtained from *Pleurotus ostreatus* cultivation residues generated as waste from shimeji production in solid-state fermentation after harvest of this edible mushroom. These residues were kindly provided by Toyobo do Brasil LTDA. 

The aforementioned residues were homogenised and kept at 28°C for twenty days before extraction. Extraction of 20 g of residue was carried out under agitation during 15 minutes using 100 mL of distilled water, 100 mL of 0.02 M sodium-tartaric buffer pH 4.0, or 100 mL of 0.02 M sodium-phosphate buffer pH 6.0. The crude extracts obtained were filtered on filter paper (Whatman no. 2) and then divided and kept at −18°C or 4°C to evaluate enzyme stability during storage. The remaining activities of laccase were monitored during 130 days. Standard deviations were less than 10%.

### 2.3. Enzyme Assays

Laccase activity was determined spectrophotometrically according to a modified method of Niku-Paavola and coworkers in 1990 [27], by monitoring the oxidation of ABTS at room temperature without agitation. The assay mixture contained 3 mM of ABTS in 0.2 M sodium-succinic buffer (pH 4.5) and 100 *μ*L of laccase rich extract from *P. ostreatus* or commercial laccase in appropriate dilution in a final volume of 2 mL. Oxidation of ABTS was monitored at 436 nm (*ξ*_436_ = 29300 M^−1^ cm^−1^). 

Enzymatic activity of oxidases, which depend or not on peroxide, was also evaluated by using 2,4-dichlorophenol and 4-aminoantipyrine as substrates [28]. Oxidation reaction was measured at 510 nm during 90 seconds (*ξ*_510_ = 18500 M^−1^ cm^−1^). Enzyme activity was expressed in International Units (IUs) as the amount of enzyme required to release 1 *μ*mol of product in 1 minute.

The presence of cellulases and proteases activities was also investigated [29, 30]. These measurements were performed in the Laboratory of Enzyme Technology—Chemistry Institute of Federal University of Rio de Janeiro. Cellulases were measured because *P. ostreatus* was cultivated using sawdust as support. Proteases were also determined to evaluate the influence of proteases in laccases activity. The presence of cellulases and proteases was not found.

### 2.4. Comparative Study between the Crude Extract and Commercial Laccase

The decolourisation of four reactive dyes was performed using crude extract and commercial laccase Novozym 51003. 

Decolourisation reactions were carried out in a wide range of dye concentrations: 60, 120, and 240 ppm. The laccase activity in the reaction media was 0.02 IU/mL and, when it was necessary, 0.017 mM of ABTS mediator was used. All reactions were performed at pH 4,0 (sodium tartarate buffer 0,2 M) without agitation. No changes in pH were observed during reactions. Dye decolourisation was measured spectrophotometrically after 1 and 24 h of reaction at the corresponding maximum absorption wavelength for each compound (DMBLR—616 nm; DMBBLN—626 nm; DMR—530 nm; RBBR—602 nm). All dyes concentrations were tested in similar conditions. 

Additional study considering the decolourisation of a mixture of these four textile dyes was performed. In this case, the concentration of each dye was 60 ppm, and the crude extract in pH 4.0 or commercial laccase was used in the absence (E) or presence (EM) of 0.017 mM of ABTS. Laccase activity in reaction media was 0.016 IU/mL. Degradation experiments were done in duplicate, and average values were reported.

## 3. Results

### 3.1. Enzyme Extraction from Shimeji (*P. ostreatus*) Residues

Similar laccase activities (255 ± 5 IU/mL) were found in the enzyme extraction from solid state fermentation residues using buffers or distilled water. Some hydrolases were also monitored, but only laccase showed expressive activity.

**Figure 1:**
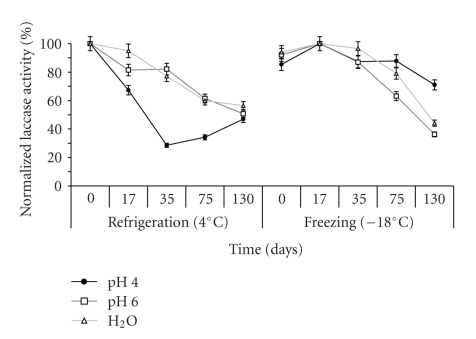
Laccase activity profile during 130 days. Extracts prepared in pH 4.0, pH 6.0, or distilled water preserved under refrigeration or freezing.

Figure 1 shows the laccase activity profile under refrigeration (4°C) or frozen (−18°C) during the storage study for 130 days. Extracts in pH 4.0, pH 6.0, and H_2_O, kept at 4°C, showed 47, 51, and 56% of remaining activities, respectively. The best stability result at −18°C was found in the laccase rich preparation extracted with buffer pH 4.0, as no significant activity variation was detected after 75 days, and about 70% of the activity remained after 130 days. Frozen extracts in pH 6.0 and H_2_O showed remaining activities of 36 and 44%, respectively.

Considering the enzyme extraction, preliminary results showed that the extracts obtained at pH 6.0 or distilled water presented lower decolourisation results compared to the crude enzyme extracted at pH 4.0 (data not shown). Considering the aforementioned results, the subsequent experiments for dye decolourisation were carried out using the laccase rich extract obtained at pH 4.0.

The stability of the ligninolytic complex is considered to be one of the determinant factors for the technical and economical viability of its industrial application to degradation of pollutants, as well as for the optimization of commercial enzyme production [31]. Therefore, the maintenance of enzymatic activity during storage is an important aspect. Previous studies [14] detected only 30% of remaining activity in the extracts kept frozen after a 30-day period, and the extracts stored under refrigeration showed a progressive activity loss during an 80-day period. Differently from our work, the extracts obtained by Machado and Mateus presented higher stability under refrigeration [14]. These authors also observed an increase on the laccase activity in some extracts during the refrigeration or freezing preservation and suggested that the existence of inhibitory substances, only present in the initial phase after extraction, could justify this increase on the activity, which corroborates the results achieved in our stability studies. The ligninolytic pool obtained by Machado and Matheus [14] from *Pleurotus ostreatus* presented higher peroxidases activities, and these peroxidases were also responsible for RBBR decolourisation. In the present work, only laccases showed significant activities, and it is important to mention that different parts and different times of the cultivation were approached in each study, demonstrating the potential source of enzymes.

### 3.2. Dye Oxidation Catalysed by Laccases

All reactions were carried out in pH 4.0 in accordance with preliminary results and literature [14, 18].  Table 2 summarizes the percentages of colour removal obtained employing reactive dyes DMBLR, DMBBLN, DMR, and RBBR in the following concentrations: 60, 120, and 240 ppm, using enzymatic extract of *P. ostreatus* in pH 4.0 in the absence (E) or presence of ABTS mediator (EM). The maximum wavelength of each dye was monitored during all reactions, and the decolourisation percentage was determined by the difference from initial and final absorbance. The decolourisation of DMBLR was observed in all concentrations studied, achieving values higher than 85% in 1 h and 97% in 24 h of reaction in the absence of ABTS mediator. Similar results were found for DMBBLN, solutions with 80% of decolourisation were obtained after 24 h also in the absence of ABTS. These results indicated that laccases produced by *P. ostreatus* are efficient in colour removal of DMBLR and DMBBLN solutions in the absence of ABTS mediator.

On the other hand, the use of ABTS mediator was essential in the DMR decolourisation. It was possible to achieve more than 86% of DMR colour removal after 1 h in concentrations lower than 240 ppm. The presence of ABTS also increased the decolourisation of RBBR by the laccase rich extract. The colour removal was improved from 61% to 90% and from 57% to 82% after 24 h using dye concentrations of 60 and 120 ppm in the absence and presence of ABTS, respectively. 

Table 3 summarizes the decolourisation values of reactive dyes DMBLR, DMBBLN, DMR, and RBBR, in the following concentrations: 60, 120, and 240 ppm, using commercial laccase in the absence (E) or presence of ABTS mediator (EM). In the DMBLR decolourisation reaction, the presence of ABTS was not necessary. This dye showed more than 95% of colour removal after 1 hour. The presence of ABTS was fundamental for the DMR and RBBR. The colour removal of DMR dye incubated for 1 hour with commercial laccase was higher than the 80% at 60, 120, and 240 ppm. Similar results were found testing RBBR dye; however, it was necessary to have 24 h of incubation. About 80% of DMBBLN decolourisation was achieved using both enzymes preparations, even in the presence of the ABTS mediator.

Tables 2 and 3 show the percentage of colour removal of reactive dyes DMBLR, DMBBLN, DMR, and RBBR (60 ppm) in the presence of commercial laccase (*A. oryzae*) and laccase rich extract (*P. ostreatus*) in pH 4.0. Both enzymes preparations are able to catalyse the decolourisation of the dyes studied, but in several cases, DMBBLN presented relevant decolourisation using the laccase rich extract after 24 h, around 80% of colour removal, while the commercial laccase did not provide satisfactory decolourisation percentage (approximately 60% in 24 h). DMBLR decolourisation with commercial laccase or laccase rich extract presented the best percentage of decolourisation, more than 95% in only 1 h. The presence of ABTS was essential for the DMR and RBBR. The colour removal of DMR dye incubated for 1 hour with the crude extract or commercial laccase was higher than 80%. RBBR showed higher values of colour removal (above 90%) in 24 h, corroborating previous works [14, 24].

Many studies have shown that fungi or their enzymes are able to decolourise and detoxify industrial dyes [5, 15, 23]. It is not novel that *P. ostreatus* produces laccases effective for decolourisation of reactive dyes; however, the value of this report lies in the identification of significant activity in agrowaste from commercial production of edible mushroom shimeji. In general, dyes decolourisation catalysed by crude extracts or purified laccases obtained from white-rot fungi has been most efficient using pH ranging from 3.0 to 5.0 [32]. Michniewicz and coworkers [23] investigated the pH effects in the decolourisation of Acid Blue 62, Acid Blue 40, Reactive Blue 81, Direct Black 22, and Acid Red 27, using laccases isoforms from *Cerrena unicolor*. Laccase I isoform kept 75% of activity in the pH range of 2.5 to 5.0, while laccase II isoform presented higher activity in pH 3.0, decreasing its activity as pH values increased. Machado and Matheus [14] carried out experiments of RBBR decolourisation using *P. ostreatus *crude extract, cultivated for 25 days, aiming to evaluate the most suitable pH ranging from 3.0 to 7.0. The best decolourisation results were achieved at pH 4.0, corroborating the results presented in Tables 2 and 3.

The dye RBBR has been widely used as model compound in decolourisation studies [14]. Previous studies developed in our research group (data in press) also showed a very promising action of horseradish peroxidase. This plant enzyme was able to remove 95% of the RBBR colour in a solution containing 120 ppm of this dye in 5 minutes of reaction.

Palmieri and coworkers [16] reported that RBBR decolourisation mediated by extracellular enzymes from *P. ostreatus* was dependent on reaction media conditions such as temperature, pH, and enzyme concentration. The fungi *P. ostreatus* was able to decolourise more than 90% of RBBR with 3 days at low (5 *μ*M) and with 6 days at high (50 *μ*M) dye concentration. These authors also observed a reduction in toxicity of 95% after decolourisation reactions considering the inhibition of the bacterium *Bacillus cereus* in the presence of the dyes before and after treatment. 

RBBR decolourisation was also studied using laccases from *Trametes versicolor* and HBT as mediator [24]. In this study, 40% of RBBR decolourisation was achieved applying free laccases and HBT after 30 minutes and 70% of RBBR decolourisation after 2 h, with immobilised laccases.

Özsoy and coworkers [21] compared Drimaren Blue X3LR (DMBLR) and Remazol Brilliant Blue R (RBBR) degradation mediated by two different fungi, *Phanerochaete chrysosporium* and *Funalia troggi*, and demonstrated that colour removal efficiency varies according to the microorganism. *P. chrysosporium* provided 11–20% of decolourisation after 10 days, while *F. troggi* was able to decolourise 92–98% of both dyes in a period of 4–10 h. Erkut and coworkers [33] also reported the differences found among *Funalia trogii, Coriolus versicolor, *and* Pleurotus ostreatus* in decolourisation reactions of Drimaren Blue CL- BR (DB) and Remazol Brilliant Blue R (RBBR) under static conditions, with 30°C of temperature and pH 5.0; *F. trogii* removed about 90% of colour of both RBBR and DB dyes after 48 h.

Ciullini and coworkers [34] studied decolourisation levels of six different classes of dyes. In all reaction media, the concentration of the different dyes was 500 mg/L, and laccase activity was kept equal to 1.5 IU/mL. Chromocomplexed azo, diazo, and anthraquinonic dyes presented percentages of colour removal higher than 85%; however, monoazo and diazo achieved only 17–28%. Other studies emphasizing the influence of chemical structure in the recalcitrance of certain dyes have been well documented. Nozaki and coworkers [35] tested 27 dyes from 6 different classes and showed the difference of two monoazo dyes decolourisation with similar chemical structures of Acid orange 20 (AO20) and 7 (AO7). The first presented about 71–100% of decolourisation and the second only 20%. 

Hou and coworkers [36] studied the decolourisation of an antraquinone dye also using a crude extract from *P. ostreatus *(strain 32). The dye concentration in the reaction mixture was 100 ppm, pH 4.5, at temperature of 50°C and 30 IU/mL of laccase activity (in terms of ABTS substrate). The results showed that 70% of decolourisation was obtained when only the crude extract was employed and 90% using crude extract in the presence of 0.16% of ABTS after 5 h of reaction. 

Comparing the colour removal results using laccase rich extract from *P. ostreatus* residues and the aforementioned results from literature, it was possible to obtain a close or high decolourisation percentage of the studied dyes. The reaction media employed in the present work contained only 0.02 IU/mL of laccase activity, approximately 30 times lower than laccase activity values reported by previous studies [8, 16, 24, 35]. Our work emphasized the use of a temperature close to room temperature, that is, 35°C, in order to minimise energy consumption. We also used a range of dyes concentrations from 60 to 240 ppm, and even acting in a concentration 4 times higher than the initial concentration (60 ppm), the colour removal of the studied dyes was not affected, indicating the enzyme efficiency in a broad range of dye concentration.

### 3.3. Enzymatic Decolourisation of a Mixture of Dyes

**Figure 2:**
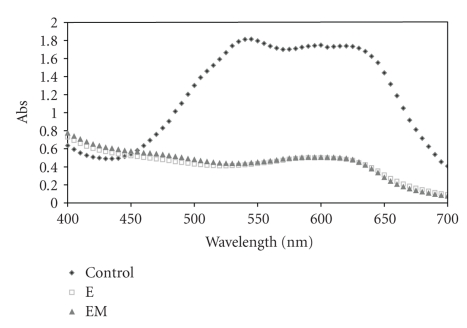
Absorption spectra of the mixture of the four dyes studied DMBLR, DMBBLN, DMR, and RBBR (60 ppm) before and after decolourisation reaction with crude extract in the presence (EM) and absence (E) of ABTS mediator.

**Figure 3:**
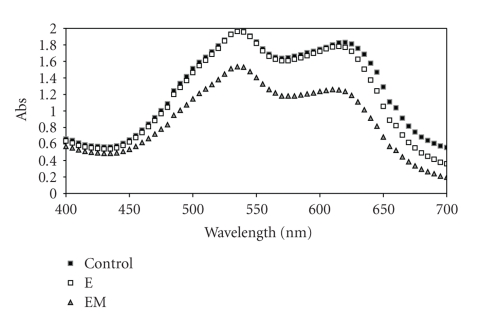
Absorption spectra of the mixture of the four dyes studied DMBLR, DMBBLN, DMR, and RBBR (60 ppm) before and after decolourisation reaction with commercial laccase in the presence (EM) and absence (E) of ABTS mediator.

In general, effluents from textile industries contain a mixture of dyes and auxiliary substances used during the dyeing process. The decolourisation of a mixture containing the four textile dyes was also evaluated. The absorption spectrum of the reaction mixtures containing 60 ppm of each dye, with the laccase rich extract in the absence (E) or presence of ABTS (EM), and with the commercial laccase in the absence (E) or presence of ABTS (EM), was presented in Figures 2 and 3, respectively.

The laccase rich extract was able to perform a more effective decolourisation (Figure 2) when compared to the commercial laccase (Figure 3). The decolourisation of the mixture with crude extract, with or without ABTS, was higher than 70% in the maximum absorption. 

Although the commercial laccase was effective in the decolourisation of DMBLR, DMR, and RBBR, the results of colour removal in the dye mixture showed a lower percentage when compared to the crude extract. This result could be explained by the occurrence of some interactions among the dyes or probably by the remaining DMBBLN contribution.

## 4. Conclusions

The present study showed the utilization of an agroindustrial residue to obtain a product with a high added value. This procedure also minimizes industrial disposal. The laccase rich extract presents an enzymatic pool with broad catalytic action, and it was prepared without purification or stabilization steps in order to obtain a low-cost biocatalyst. The decolourisation efficiency of reactive dyes in the presence of the laccase rich extract was similar or even better than the commercial laccase although we used a laccase activity 30 times lower than the values previously reported in the literature.

Important results related to reactive dyes decolourisation were obtained, and the use of ABTS was relevant only for the decolourisation of DMR and RBBR dyes using both laccases. Although the temperatures used in decolourisation reactions reported in the literature vary from 40 to 50°C, the temperature of 35°C, which was applied during our studies, proved to be efficient as well. This data is also important to preserve or minimize energy consumption. The enzymatic extract from *P. ostreatus* was more efficient in the decolourisation of the mixture of dyes than the commercial from A. oryzae. 

Our work has demonstrated that the use of an industrial waste to obtain an enzymatic complex could be economically interesting and also contribute with an ecofriendly alternative. Future studies should test this enzymatic pool combined with other types of treatment to remove colour from textile industries wastewater.

## Figures and Tables

**Table 1:** Chemical structure and properties of the four reactive dyes studied.

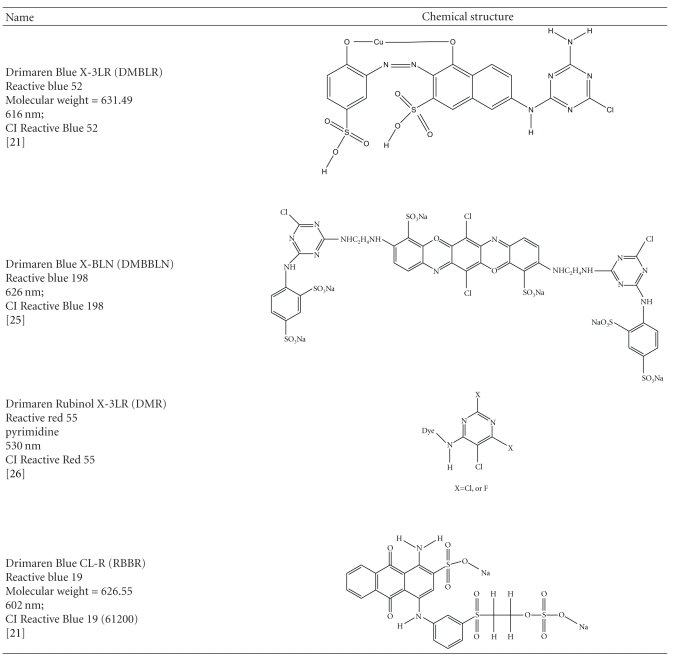

**Table 2:** Colour removal of the reactive dyes DMBLR, DMBBLN, DMR, and RBBR, after 1 h and 24 h, in the presence of the crude enzymatic extract from shimeji (*P. ostreatus*) residues obtained in pH 4.0 in the absence (E) or presence of ABTS mediator (EM). In bold the percentage of decolourisation over 70%.

Dyes	Concentration (ppm)	Colour Removal (%)			
		Without ABTS		With ABTS	
		1 hour	24 hours	1 hour	24 hours
DMBLR	240	**96.99**	**98.84**	**96.72**	**98.60**
	120	**96.59**	**99.15**	**93.50**	**98.14**
	60	**95.82**	**99.48**	**87.06**	**97.19**

DMBBLN	240	68.02	**82.01**	66.06	**80.68**
	120	68.34	**83.62**	65.96	**79.43**
	60	66.26	**83.97**	57.54	**74.11**

DMR	240	26.70	66.03	**86.33**	**94.07**
	120	27.88	69.14	**94.97**	**93.46**
	60	38.35	66.25	**92.56**	**91.04**

RBBR	240	23.97	57.14	33.35	**72.47**
	120	33.71	57.51	36.16	**82.30**
	60	36.29	61.20	36.68	**90.17**

**Table 3:** Colour removal of the reactive dyes DMBLR, DMBBLN, DMR, and RBBR, after 1 h and 24 h, in the presence of the commercial laccase from *Aspergillus orizae* in the absence (E) or presence of ABTS mediator (EM). In bold the percentage of decolourisation over 70%.

Dyes	Concentration (ppm)	Colour Removal (%)			
		Without ABTS		With ABTS	
		1 hour	24 hours	1 hour	24 hours
DMBLR	240	**97.50**	**98.90**	**96.10**	**98.00**
	120	**97.00**	**98.70**	**92.40**	**96.60**
	60	**96.90**	**98.90**	**84.70**	**93.30**

DMBBLN	240	30.60	57.40	32.40	59.30
	120	31.20	59.80	31.00	61.80
	60	31.70	63.00	23.80	62.90

DMR	240	0.30	1.00	**79.70**	**91.70**
	120	3.50	3.50	**90.50**	**89.70**
	60	1.00	3.30	**84.90**	**87.90**

RBBR	240	29.70	44.00	48.00	**79.90**
	120	33.00	57.10	**72.30**	**94.40**
	60	31.00	64.40	**74.20**	**95.70**
